# Specific IgE and skin prick tests to diagnose allergy to fresh and baked cow’s milk according to age: a systematic review

**DOI:** 10.1186/s13052-017-0410-8

**Published:** 2017-10-12

**Authors:** Barbara Cuomo, Giovanni Cosimo Indirli, Annamaria Bianchi, Stefania Arasi, Davide Caimmi, Arianna Dondi, Stefania La Grutta, Valentina Panetta, Maria Carmen Verga, Mauro Calvani

**Affiliations:** 1 Operative Complex Unit of Pediatrics, Belcolle Hospital, Viterbo, Italy; 2 Operative Unit of PediatricAllergy, San Giuseppe da Copertino Hospital, Copertino, LE Italy; 3 Operative Complex Unit of Pediatrics, S. Camillo-Forlanini Hospital, Rome, Italy; 4 Department of Pediatrics, University of Messina, Messina, Italy; 5 Allergy Unit of the Department of Respiratory Diseases, University Hospital of Montpellier, Montpellier, France; 6 Sorbonnes Universités, UPMC Paris 06, UMR-S 1136, IPLESP, Equipe EPAR, F-75013 Paris, France; 7 Department of Pediatric Emergency, S.Orsola-Malpighi Hospital, Bologna, Italy; 8 Institute of Biomedicine and Molecular Immunology, National Research Council, 90100 Palermo, Italy; 9 L’altrastatisticasrl, Consultancy & Training, Biostatistics office, Rome, Italy; 10 Primary Care Pediatrics, ASL Salerno, Vietri sul Mare, SA Italy

**Keywords:** Children, Cow’s milk allergy, Cut-offs, Predictive value, Skin prick test, α-lactalbumin, β-lactoglobulin, Casein, Positive predictive value, Specificity, Oral food challenge

## Abstract

**Background:**

The diagnosis of IgE-mediated cow’s milk allergy is often based on anamnesis, and on specific IgE (sIgE) levels and/or Skin Prick Tests (SPT), which have both a good sensitivity but a low specificity, often causing positive results in non-allergic subjects. Thus, oral food challenge is still the gold standard test for diagnosis, though being expensive, time-consuming and possibly at risk for severe allergic reactions.

**Aim:**

The aim of the present study was to perform a systematic review of the studies that have so far analyzed the positive predictive values for sIgE and SPT in the diagnosis of allergy to fresh and baked cow’s milk according to age, and to identify possible cut-offs that may be useful in clinical practice.

**Methods:**

A comprehensive search on Medline via PubMed and Scopus was performed August 2017. Studies were included if they investigated possible sIgE and/or SPT cut-off values for cow’s milk allergy diagnosis in pediatric patients. The quality of the studies was evaluated according to QUADAS-2 criteria.

**Results:**

The search produced 471 results on Scopus, and 2233 on PubMed. Thirty-one papers were included in the review and grouped according to patients’ age, allergen type and cooking degree of the milk used for the oral food challenge.

In children < 2 years, CMA diagnosis seems to be highly likely when sIgE to CM extract are ≥ 5 KU_A_/L or when SPT with commercial extract are above 6 mm or Prick by Prick (PbP) with fresh cow’s milk are above 8 mm. Any cut-offs are proposed for single cow’s milk proteins and for baked milk allergy in children younger than 2 years. In Children ≥ 2 years of age it is hard to define practical cut-offs for allergy to fresh and baked cow’s milk. Cut-offs identified are heterogeneous.

**Conclusions:**

None of the cut-offs proposed in the literature can be used to definitely confirm cow’s milk allergy diagnosis, either to fresh pasteurized or to baked milk. However, in children < 2 years, cut-offs for specific IgE or SPT seem to be more homogeneous and may be proposed.

## Background

Cow’s milk (CM) is one of the first causes of food allergy in the first years of life [1] and of food anaphylaxis in pediatric patients [2]. Cow’s milk allergy (CMA) has a prevalence ranging between 1.8 and 7.5% in the first year of life [3]. CMA diagnosis is often based on a compatible clinical history and on the results of specific IgE (sIgE) and/or skin prick tests (SPT). Specific IgEs and SPTs to CM extract or to the single CM allergenic proteins show a good sensitivity but a low specificity. Therefore, sensitization does not correlate well with allergy [4]. If the diagnosis of CMA were only based on sIgE or SPT results, a group of sensitized but non-allergic subjects would uselessly undergo a CM-exclusion diet. Hence, Oral Food Challenge (OFC) is still considered as the gold standard for CMA diagnosis, despite being expensive, time-consuming, and possibly causing allergic reactions which may even result in anaphylaxis.

It has been shown that, the greater the food-sIgE levels and the SPTs wheal size, the higher the chances that patients react during an OFC [4]. This is the reason why some authors have investigated if it is possible to establish a cut-off for sIgEs and SPTs to CM or its proteins, that could predict by itself whether a patient would react to an OFC. Several studies showed that cut-offs may vary with age [5], and previous reviews proposed practical indications to diagnose of food allergy and suggest different diagnostic cut-offs for children, based on age [6–8]. However, cut-offs may vary also because of the cooking degree [9] or the type of allergen used to perform SPTs (commercial extract vs. raw milk). Thus, in the present Systematic Review, we grouped studies according to these three factors.

The aim of this study was to compare, in children with suspected CMA, the levels of sIgEs and the wheal sizes of SPTs for CM or its three main allergenic molecules (α-lactalbumin (αLA), β-lactoglobulin (βLG), and casein) with the Reference Standard (RS) test, OFC, in order to identify any validated cut-off value. We analyzed available data from a methodological point of view and tried to provide practical clinical indications for the diagnosis of CMA in children. At the best of our knowledge, such a classification has never been considered in previous studies [6–8].

## Methods

### Inclusion and exclusion criteria for considering studies for this systematic review

We included studies in which authors looked for a cut-off value for SPTs or sIgEs levels for the diagnosis of CMA in children. In most cases, diagnosis was based on the results of the OFC. Studies were also considered whenever a clear relationship between CM exposure and allergic reaction was highlighted and sIgE or SPTs were carried out.

Studies were excluded if information was not specific enough for CMA, or if the Authors identified the optimal cut-off only (meaning a cut-off based on the best combination between sensibility and specificity), which does not allow to adequately select patients at high risk of reacting to the OFC.

### Types of participants

We included children with suspected CMA.

### Types of outcome measures

We searched for cut-off values for CMA diagnosis using CM extract, αLA, βLG, casein, for sIgE or SPT, and using fresh milk for PbP.

### Search methods for the identification of the studies

On August 2017, we performed a comprehensive search on Medline via PubMed and Scopus, by using the strings “sIgE” or “specific IgE” or “SPT” or “skin prick test” and “milk allergy” or “milk hypersensitivity”. Search was not restricted by publication type or language or study design. If any relevant paper was identified afterwards, we included it as well [3, 10, 11].

We checked reference of all included studies and reviews, for additional references as well.

### Data collection and analysis

#### Selection of the studies

For each string, two authors independently screened titles and abstracts to consider for inclusion all potential identified studies. Full texts were searched as well, to identify studies for inclusion. We resolved disagreements through discussion or, if required, by consultation with a third person. Data extraction from reports was in duplicate and in case of doubts we directly contacted the authors to obtain and confirm data. Studies were all widely discussed in detail and evaluated by the authors in a standardized and independent manner.

**Fig. 1:**
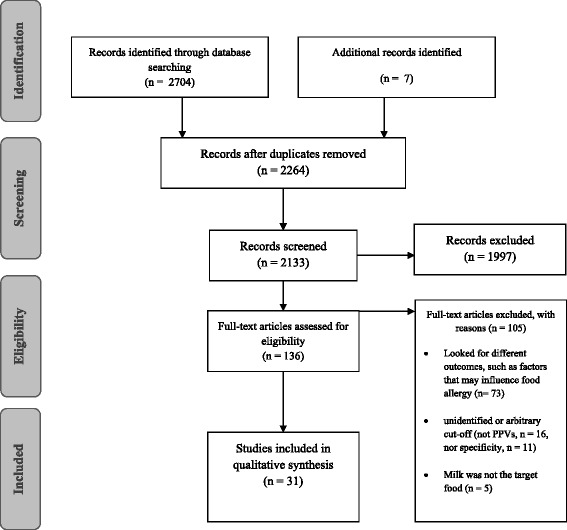
Flow chart of the search run to obtain the studies included in the present review

We recorded the selection process to complete a Preferred Reporting Items for Systematic Reviews and Meta-Analyses (PRISMA) flow diagram (Fig. 1).

#### Methodological quality evaluation of the included studies

The methodological quality of the included studies was evaluated according to criteria proposed by QUADAS-2 [12]. In order to establish the risk of bias, papers were independently revised by at least two authors, and any divergence was resolved by discussion and agreement among all reviewers.

## Results

The search identified 2233 articles of potential interest on Medline, and 471 articles on SCOPUS. After the selection process, a total of 31 articles were included in this systematic review. Of these, 22 referred to the cut-off for sIgE and 13 for SPT cut-offs (4 proposed cut-offs for both) (Fig. 1). These studies are presented separately below, grouping them based on:

1. sIgEs levels or SPT wheal size;
2. patients’ age, enrolling children:
  - < 2 years;
  - >2 years;
  - any age;
3. the cooking degree of CM administered during the OFC:
  - CM: fresh pasteurized CM (or CM formula in children <12 months of age);
  - baked milk: extensively heated CM (> 100 °C or 212 °F for several minutes).

Among the studies dealing with sIgE and SPT cut-offs, 11/22 (50%) and 7/13 (53.8%), respectively were prospective [9, 13–26], while the remaining were either retrospective or with unspecified design.

Most studies analyzed the role of sIgE and SPTs for CM in patients allergic to fresh pasteurized milk [4, 5, 13–21, 27–39]. Five studies evaluated sIgE and SPTs in patients allergic to baked milk [9, 24–26, 40].

**Fig. 2:**
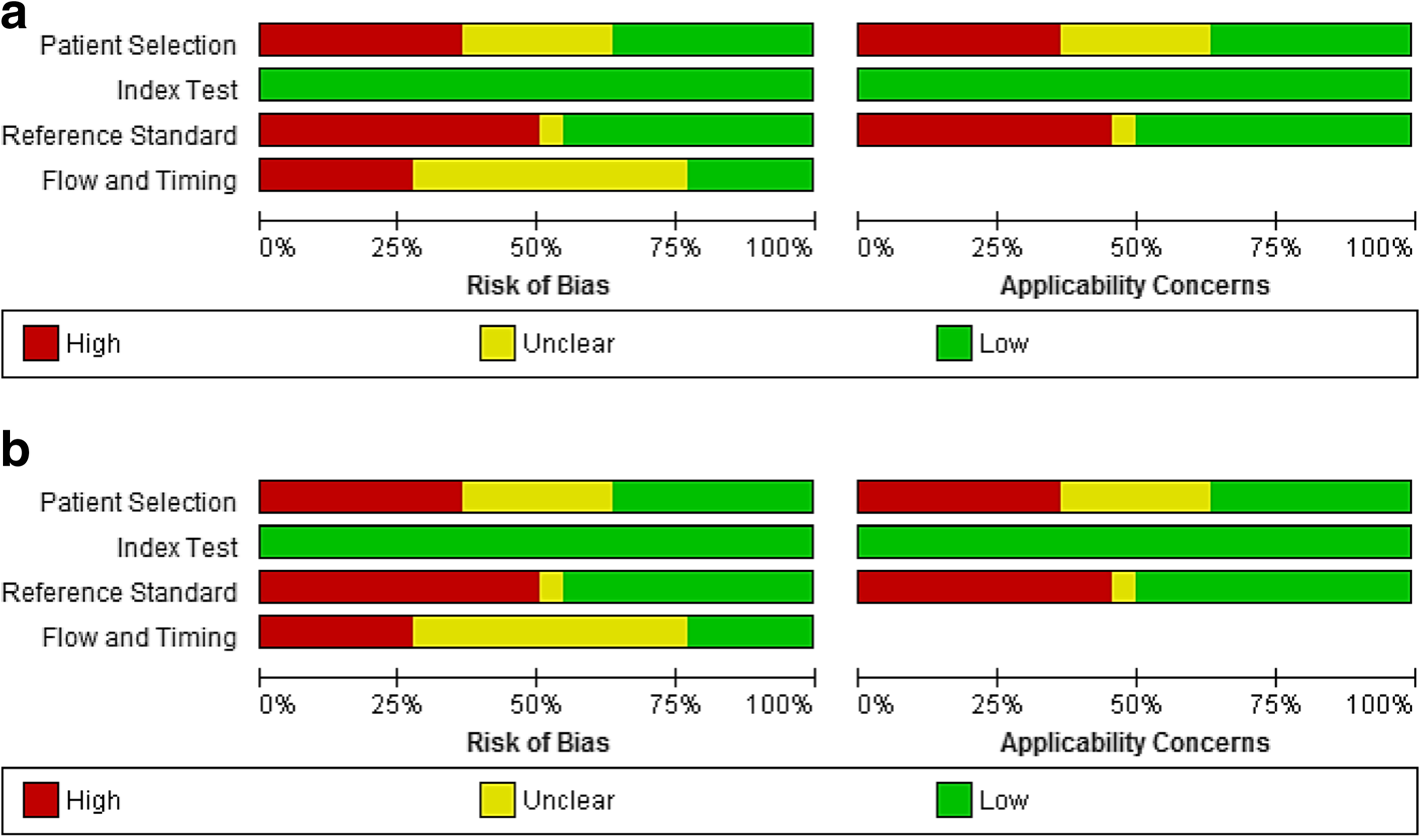
Methodological quality of the articles included in the present revision according to the QUADAS-2 tool [12]. **a** Risk of bias and applicability concerns graph: review authors’ judgements about each domain presented as percentages across included specific-IgE studies Review Manager (RevMan) [Computer program]. Version 5.3. Copenhagen: The Nordic Cochrane Centre, The Cochrane Collaboration, 2014. **b** Risk of bias and applicability concerns graph: review authors’ judgements about each domain presented as percentages across included SPT studies. Review Manager (RevMan) [Computer program]. Version 5.3. Copenhagen: The Nordic Cochrane Centre, The Cochrane Collaboration, 2014

According to QUADAS-2 evaluation: a) for sIgE studies: patients’ selection was considered at low risk for both bias and applicability in 8 studies, index test choice was at low risk for bias and applicability in all the studies, reference standard in 10 and flow and timing only in 5 (Fig. 2a; b) for SPT studies all articles but three [23, 26, 39] were judged to be at high risk of bias and applicability as for patients’ selection (Fig. 2b).

### Predictive value of sIgE and SPT for the diagnosis of fresh pasteurized CMA

Table 1 shows the 19 studies evaluating the diagnostic efficacy of CM sIgE; five of them assessed the role of sIgE for αLA, βLG, and casein as well. Studies differed in prevalence of any type of allergic disease and atopic dermatitis, statistical analysis, type of chosen cut-offs, and methodology. These factors might explain the large variability of the proposed cut-offs, which vary from 0.35 to 88.8 KU_A_/L.

**Table 1:**
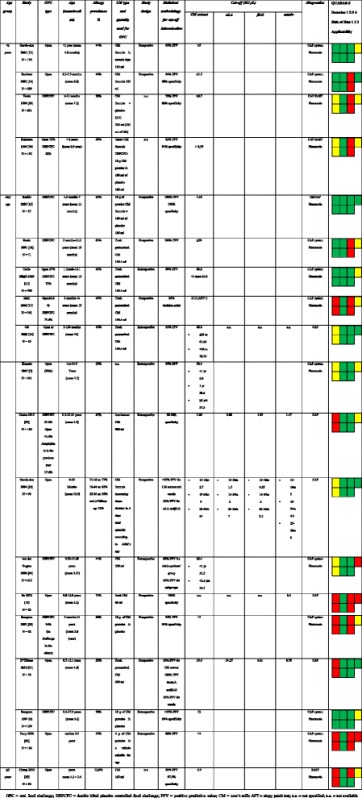
Studies and cut-offs suggested for fresh pasteurized CMA diagnosis using sIgE for CM extract, α-lactalbumin (αLA), β-lactoglobulin (βLG), and casein stratified by study design and ordered by age group [4, 5, 13–21, 29–36]

As for sIgE against CM, considering those studies including only children younger than 2 years, two prospective studies with a good QUADAS-2 evaluation and with significant patients’ numbers showed quite similar cut-offs for a 95% positive predictive value (PPV) (≥3.5 KU_A_/L [14] and ≥5 KU_A_/L [13]), even if the second cut-off was proposed in a study with an important risk of bias for its “reference standard” domain. Considering those studies including children of any age, very different values have been proposed even with similar statistical methods. For example, cut-offs with a 100% PPV varied between 4.18 KU_A_/L [15] and 50 KU_A_/L [16]. Four papers [18, 19, 21, 33] proposed sIgE cut-offs for the main allergenic CM components. These studies were conducted in children of any age and found extremely heterogeneous cut-off values, distributed in a very wide range without a clear explanation (αLA: 1.5–34 KU_A_/L; βLG: 0.35–9.91 KU_A_/L; casein: 0.78–6.6 KU_A_/L) [18, 19, 21]. The only study enrolling children aged more than 2 years had a low methodologic quality and showed a 6.9 KU_A_/L cut-off for CM extract with a 97.5% specificity [35].

Four studies evaluated PPV of SPTs through commercial extracts (Table 2a). Studies differ in allergy prevalence, statistical analysis, type of cut-off, and type of allergen used for SPTs. All these factors may help explain the variability of the cut-offs proposed by the Authors, ranging from 4.3 to 20 mm. In the paper by Calvani et al. [27], the Authors suggest that the positivity for all the three milk proteins has a higher diagnostic value (PPV > 90%) rather than the single possible cut-offs for each one of them, separately considered; a similar hypothesis has been later proposed by two more studies, even though with a lower PPV (86.7% [28] and 74% [23]).

**Table 2:**
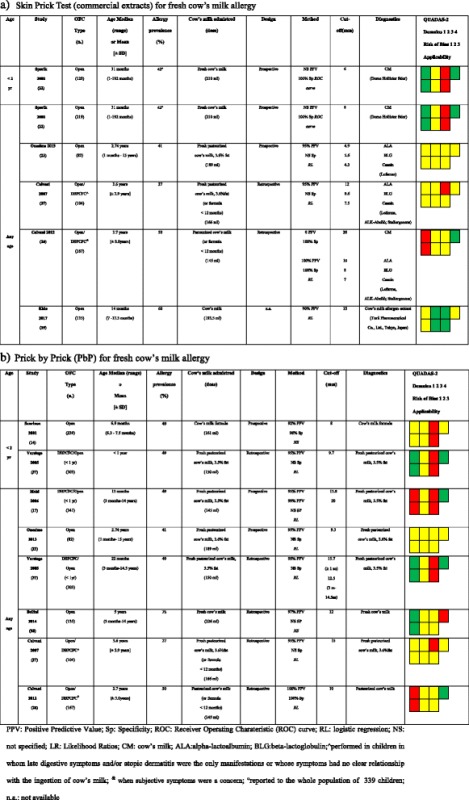
Studies and suggested cut-offs for fresh cow’s milk allergy diagnosis using alpha-lactoalbumin, beta-lactoglobulin, casein, cow’s milk SPTs stratified by the type of allergen used to perform SPTs, design, and age (<2 years and ≥2 years) [14, 17, 22, 23, 27, 28, 37–39]

Two papers evaluated a possible cut-off using PbP for CMA in children younger than 2 years [14, 37] and the results are quite similar: 8 and 9.7 mm, with a PPV of 92% and 95%, respectively. Six studies, conducted on children but with no respect to age groups, reported results that ranged between 9.3 mm and 15.7 mm (Table 2b) [17, 23, 27, 28, 37, 38].

### Predictive value of sIgE and SPT for the diagnosis of baked CMA

Three studies analyzed the diagnostic efficacy of sIgE against CM or its allergenic proteins in patients allergic to baked milk (Table 3a) [9, 24, 40]. All these studies enrolled children aged more than 2 years. The cut-offs highlighted in these papers cannot be compared due to the different statistical methods used by the Authors: e.g. for sIgEs against CM extract, Nowak-Wegrzyn proposed a cut-off of 35 KUA/L with a 85.7% PPV, whereas Caubet of 24.5 KUA/L with a 95% specificity.

**Table 3:**
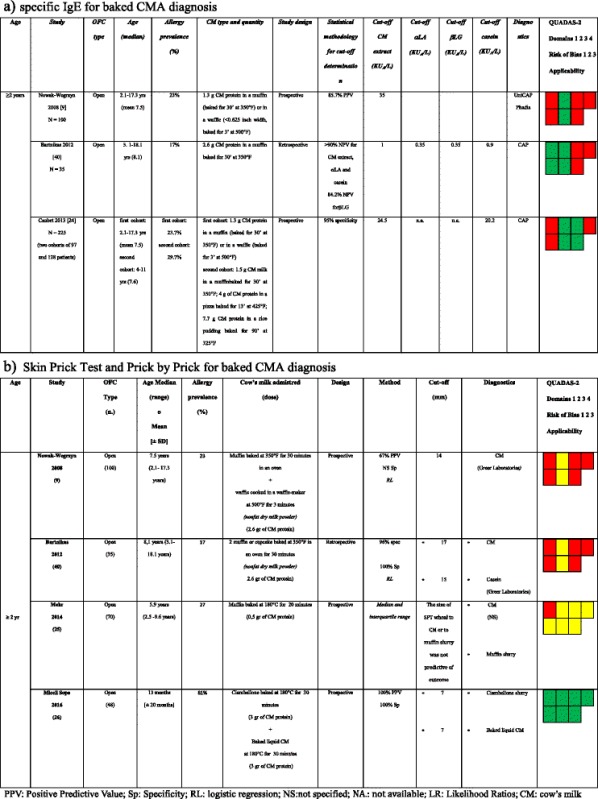
Studies and cut-offs suggested for baked CMA diagnosis using CM extract, α-lactalbumin (αLA), β-lactoglobulin (βLG), and casein for sIgE or SPT and using fresh milk for PbP. OFC = oral food challenge; PPV = positive predictive value; NPV = negative predictive value; CM = cow’s milk; n.a. = not available [9, 40, 24]

Three studies investigated a possible cut-off for SPTs using milk extracts or its proteins to diagnose allergy to milk that was extensively cooked in a grain matrix (muffins). Only two of these studies reached a conclusion (Table 3b) [5, 20, 36]. The two identified cut-offs, using CM extract, are much higher if compared with those for fresh pasteurized milk (14 and 17 mm, respectively), but differ greatly in terms of predictability (a 67% PPV in one study and a 96% specificity in the other one). Moreover, these studies showed that wheals of 5 mm and 7 mm, respectively, for CM extracts have a 100% negative predictive value (NPV). Two studies [25, 26] evaluated the predictive value of PbP with muffin or with Italian cake (named ciambellone) containing baked CM within a wheat matrix. In the first study, the size of the SPT wheal to CM or to muffin slurry was not predictive of outcome. In the second, OFCs were always failed if PbP mean wheal diameter using baked cake or baked liquid cow’s milk were >7 mm (100% PPV). The same study showed that every negative PbP corresponded to a passed OFC for baked cake in CMA patients [26].

## Discussion

Over the last years, several studies have looked for cut-offs for sIgE or SPTs able to predict CMA without the need to perform an OFC.

To find more homogeneous cut-offs, we grouped the studies according to:

1. patients’ age. Most of the studies on fresh pasteurized CMA diagnosis included children aged from few months to several years. Only the paper from Chung [35] enrolled children aged more than 1 year (mean age 3.1 ± 1.4). On the contrary, all the studies on baked CMA enrolled children aged more than 2 years. Therefore, we divided the studies into three groups: a) those enrolling children aged less than 2 years (< 2 years group); b) those enrolling children aged more than 2 years (> 2 years age group); c) and those enrolling children of any age group;
2. type of allergen. Several studies showed that SPT mean wheal diameter is usually different between commercial extracts and fresh food [27, 41];
3. cooking degree of the milk. It is well known that CM proteins are modified by exposure to high temperatures, which not only modify the conformational epitopes, but partly the sequential ones as well. Heating is one of the most common technological treatment applied to milk processing and it may have different effects on the binding of IgE to proteins. Mild treatments are not sufficient to reduce the allergenicity of milk as it has been shown for pasteurized milk, which is able to elicit allergic responses in milk allergic patients [42]. On the other hand, when milk is exposed to higher temperatures and for a longer time, its allergenicity is reduced. Moreover, when milk is cooked in a grain matrix for a long time, as it happens in baked products, its proteins are modified both by heat and by chemical reactions occurring between the matrix fats and sugars, and are therefore less likely to be recognized by the immune system of the allergic patient (the so-called “matrix effect”) [43].

### Limitations

Grouping studies has reduced the variability of the cut-offs proposed, but not substantially. On the other hand, many other factors may influence the cut-offs, both for sIgE and for SPT, such as:

- different statistical methods (e.g. PPV or specificity). Two different kinds of cut-offs values are proposed in literature, both for SPT and for sIgEs: those based on a high PPV (95% PPV) and those based on a high specificity (95% specificity). The first ones, being based on the predictive value, depend on several factors, above all on the prevalence of allergy in the studied population, background history, sex, etc., and are applicable in allergy centers where it is assumed that the diagnostic criteria and the prevalence of food allergy are similar to those found in those studies providing the values. On the contrary, cut-off values based on 95% specificity do not change with the prevalence of the disease in the population and give us the possibility to better select children to test with OFC, given the high risk of a positive challenge. These two kinds of cut-off values may produce different results even in the same study population [44].
- variations in the chosen level of predictive value in different studies (e.g. 90% vs. 95%) may substantially change the proposed cut-offs.
- methodological quality (e.g. studies with a small number of DBPCFC performed, with high risk of bias or including a small number of patients); d) differences in patients’ selection or in the definition of a positive OFC (e.g. one study [14] considers as positive an OFC in which late reactions appear at home, such as atopic dermatitis or others). Moreover, several variables may affect the wheal size of positive SPT, such as type of devices or test technique, composition and potency of commercial extracts, and the “histamine skin reactivity” [45, 46].

Finally, wheal dimensions vary widely, depending on the individual characteristics, geographical setting and may change over time [47].

### Practical clinical indications

Given the large variability of the proposed cut-offs, it is hard to propose practical clinical indications. However:

- in children < 2 years, proposed cut-offs seem to be homogeneous enough. The studies with the highest methodological quality suggest a 95% PPV cut-off for sIgEs of 5 KU_A_/L [13] and a 98% specificity cut-off for PbP with fresh milk of 8 mm [14]. As for SPT with commercial extract, the only included study, which is prospective but with QUADAS-2 bias, proposed a 100% specificity SPT cut-off of 6 mm [22]. None of the studies proposed cut-offs for single CM protein SPT and one study only did for sIgE [18]. None of the studies for baked milk allergy enrolled children aged less than 2 years;
- in children ≥2 years of age, it is hard to define practical cut-offs for CMA. The cut-offs proposed for SPT with commercial extracts or fresh milk are heterogeneous, probably because most of the studies included children of any age and with no differentiation in age groups. A large variability in cut-offs has been recorded for single CM proteins as well, especially for sIgE levels, even when selecting methodologically valid studies using the same statistical methods. For example, two DBPCFC prospective studies [15, 16], with similar allergy prevalence (respectively 62% and 63%), and similar population age (respectively 1.5 months −7 years, mean 11 months; and 11 months – 11.2 years, mean 13 months), proposed sIgE cut off values with a 100% PPV for 4.18 and >50 KU_A_/L, respectively. CM type and quantity used for OFC or other known factors listed before (e.g. methodological quality) or unknown issues could explain these differences. As for baked milk allergy, there are only a few studies investigating cut off values for both specific IgE and SPT, and they showed a low methodological value. However, using CM extract, cut-offs seem to be higher if compared with those for fresh pasteurized milk. A single prospective study with a low risk of bias and applicability showed a 100% PPV for wheal diameter cut-off value of 7 mm when fresh CMA patients were pricked with baked cake for predicting baked CMA [26].

## Conclusions

No proposed cut-off can be used to definitely confirm a diagnosis of CMA, either with fresh pasteurized or with baked milk. Cut-offs may be affected by many factors, and especially PPV cut-offs may be considered as useful only in the same allergy unit in which they were detected, and may be extrapolated to other centers only if they have similar allergy prevalence. However, with these limits, in children < 2 years, when sIgE against CM are above 5 KU_A_/L or when SPT with commercial extract are above 6 mm or PbP with CM are above 8 mm, the real need for a diagnostic confirmation of CMA through an OFC should be carefully evaluated.

## Abbreviations

CM: Cow’s milk
CMA: Cow’s milk allergy
DBPCFC: Double blind placebo controlled food challenge
NPV: Negative predictive value
OFC: Oral food challenge
PbP: Prick by Prick
PPV: Positive predictive value
sIgE: Specific IgE
SPT: Skin prick test
αLA: α-lactalbumin
βLG: β-lactoglobulin
